# Phenotypic Variability of Liberoid Coffee (*Coffea liberica* and C. *dewevrei*) Across Sumatera and Javanese Landscapes: Foundational Insights for Coffee-based Agroforestry

**DOI:** 10.21315/tlsr2026.37.3.3

**Published:** 2026-10-15

**Authors:** Danniary Ismail Faronny, Melati Julia Rahma, Ayudha Pradhana, Agus Nurrofik, Wenny Bekti Sunarharum, Luchman Hakim, Soemarno, Elexson Nillian, Tunjung Mahatmanto

**Affiliations:** 1 Environment Sciences Doctoral Program, Postgraduate School, Universitas Brawijaya, Jl. MT Haryono 169, Malang 65145, Indonesia; 2 Department of Geography, Faculty of Social Sciences, State University of Malang, Jalan Semarang 5, Malang 65145, East Java, Indonesia; 3 Department of Business Administration, Universitas Brawijaya, Jl. MT Haryono 169, Malang 65145, Indonesia; 4 Department of Biology, Faculty of Mathematics and Natural Sciences, Universitas Brawijaya, Jl. MT Haryono 169, Malang 65145 Indonesia; 5 Department of Food Science and Biotechnology, Faculty of Agricultural Technology, Universitas Brawijaya, Jl. MT Haryono 169, Malang 65145 Indonesia; 6 Department of Soil Sciences, Faculty of Agriculture, Universitas Brawijaya, Jl. MT Haryono 169, Malang 65145 Indonesia; 7 Faculty of Resource Science and Technology, University Malaysia Sarawak, 94300 Kota Samarahan, Sarawak, Malaysia

**Keywords:** Agroforestry, Environment, Liberoid Coffee, Phenotypic, Variability

## Abstract

This study reveals the phenotype diversity of Liberoid coffee in three agroforestry landscapes on two islands (Sumatra and Java) with contrasting soil conditions and microclimates, namely Betara (Jambi) in peatlands, and Wonosalam (Jombang) and Silo (Jember) in mineral soils. Principal Component Analysis (PCA) shows that primary variation is determined by the character of the fruit and seeds, while size and vegetative structure contribute as an additional source of diversity. The results of hierarchical clustering (AHC) show that the Jambi population is divided into three small groups with high heterogeneity, while most of the Javanese genotypes tend to be more homogeneous. The acidic conditions of the peat soil in Jambi encourage Liberoid coffee to adapt to the small but thick pulp fruit, while the soil in Java (pH tends to be neutral) stimulates vegetative growth more rapidly. These results confirm the urgency of preserving local coffee genetic resources for plant resilience in the face of various agroecological pressures, as well as being a foothold for the breeding of adaptive varieties based on agroforestry landscapes.

**HIGHLIGHTS**

- Climate change has significantly reduced coffee productivity and quality in Indonesia, causing shifted growing seasons, poorer seed quality and more pests and diseases
- Liberoid coffee (*C liberica* and *C. dewevrei*) is more resistant to drought, pests and diseases than Arabica and Robusta, but it has been neglected in research.
- Its potential for tropical agroforestry remains untapped because comprehensive scientific data is lacking.
- More research is needed to identify the genetic basis of its variability, link plant morphology to secondary metabolites and flavour and test how it responds to heat and pathogens on marginal lands.

## INTRODUCTION

Climate change has had a significant impact on coffee productivity in Indonesia. The phenomenon of rising average temperatures, changes in rainfall patterns, and increasing frequency of extreme weather events have disrupted the growth and yield of coffee (Girma 2023; Legesse 2019). In addition, according to the results of the study (Alotaibi 2023), changes in erratic rainfall patterns cause shifts in planting and harvesting seasons. Other data revealed that climate change also affects the quality of coffee beans (Bakri *et al*. 2018). The results of the study (DaMatta & Ramalho 2006) explain that exposure to high temperatures and stress due to drought can reduce the quality of coffee taste. Then it is also explained that an increase in extreme rainfall can cause a decrease in the physical quality of coffee beans (Kath *et al*. 2021). According to the report (Ramadhillah & Masjud 2024), climate change increases the vulnerability of coffee plants to pest and disease attacks. Where this condition exacerbates the continuous decline in coffee productivity and quality.

The need for alternative coffee varieties resilient to climatic pressures is becoming increasingly urgent, as the impacts of climate change on coffee productivity and quality become more pronounced (Ramadhillah & Masjud 2024). This underscores the importance of research into the genetic diversity of underutilised coffee species (Davis *et al*. 2022). One group demonstrating high adaptive potential is the Liberoid clade (subgenus Pachycoffea), which includes taxa such as *Coffea liberica* var. liberica, *C. liberica* var. dewevrei (commonly known as Excelsa), C*. abeokutae* and related allied species (Clifford & Willson 1985; Davis *et al*. 2022). Historically, this group was introduced from Liberia to Indonesia during the Dutch colonial period as a biological solution to the leaf rust epidemic (*Hemileia vastatrix*), leading to its popular designation as the Liberoid group (Cramer 1957). Taxonomically, it has often been synonymised under *C. liberica* var. liberica and *C. liberica* var. dewevrei (Bridson 1982). However, recent phylogenetic studies provide clearer delineation, recognising *C. liberica, C. dewevrei*, and *C. klainei* as distinct species, thereby paving the way for exploring their unique agronomic and physiological traits (Davis *et al*. 2025). Empirical evidence and prior studies indicate that Liberoid coffees possess superior adaptability to drought stress and demonstrate significant resistance to certain pests and diseases compared to *C. arabica* and *C. canephora* (Robusta) (Cramer 1957; Davis *et al*. 2022; Faronny *et al*. 2024).

Building upon its promising genetic and adaptive traits, the potential of the Liberoid clade for climate-resilient cultivation appears significant. Despite this potential, its existence has often been marginalized in both research and development compared to major commercial species such as *C. arabica* (Arabica) and *C. canephora* (Robusta). In reality, preliminary evidence suggests that Liberoid coffees possess a notable ability to adapt to extreme agroecological conditions and exhibit inherent resistance to certain diseases, making them ideal candidates for coffee-based agroforestry systems in the tropics (Hafif *et al*. 2024).

However, the translation of this potential into optimised agricultural systems is hindered by a critical lack of scientific data. Specifically, comparative studies on the phenotypic variability of Liberoid coffee across various ecosystems in landscapes of Sumatra and Javaremain strikingly limited. These regions offer a natural laboratory with significant gradients in elevation, soil types, rainfall patterns, and microclimates (Joshi 2023). The absence of research that systematically correlates the phenotypic expression of Liberoid genotypes with specific agroforestry conditions has prevented the formulation of science-based cultivation protocols, leaving their potential largely untapped within sustainable production frameworks.

Previous investigations have predominantly focused on localised, basic agronomic aspects of Liberoid coffee (Faronny *et al*. 2024; Hafif *et al*. 2024).These studies have not sufficiently addressed the broader spectrum of phenotypic diversity across environments or explicitly linked morphological traits to mechanisms of ecological adaptation. This is an important gap, especially given the potential of this species as part of adaptive solutions in the face of climate change pressures and declining conventional coffee productivity.

This research aims to address this gap by investigating the phenotypic diversity of liberoid coffee in various agroecological landscapes in Sumatra and Java, as well as evaluating how these variations can be associated with the development potential of agroforestry systems. This study will also examine the relationship between phenotypic expression and the possibility of superior genotype selection based on growth location.

This study focuses on the phenotypic diversity of Liberoid coffee across the environment in the context of agroforestry in Indonesia. In addition, the landscape and ecological approaches used can provide a new understanding of the potential for adaptation of minor coffee species in the face of extreme climate challenges.

Practically, the results of this study will contribute to the novelty of information related to conservation and breeding efforts for coffee plants based on local diversity and ecological adaptation, as well as encourage the diversification of coffee species that are more resilient to climate change. This knowledge can be used as a basis for planting material selection, adaptive coffee agroforestry design, to the development of conservation efforts and improving the welfare of farmers in marginalized areas on agroforestry land.

## MATERIALS AND METHOD

The research was conducted in three locations of Liberoid coffee agroforestry plantation owned by local farmers on the islands of Java and Sumatra, Indonesia. The Sumatran agroforestry coffee plantation observed in Batara District, West Tanjung Jabung Regency, Jambi Province (−1°1′24.68″S, 103°21′56.85″E) represents Liberoid coffee agroforestry growing on peatland. Meanwhile, the observation plantation on Java Island are located in two locations, namely Silo District, Jember Regency (–9°40′15.71″S, 113°51′54.01″E) and Wonosalam District, Jombang Regency (–8°16′25.92″S, 112°22′53.07″E), East Java Province. The research was conducted from June to November 2024. The survey and field observation areas at the three locations, namely Batara in Sumatra and Wonosalam and Silo in Java show in Fig. 1, represent different environmental and topographical conditions, as reflected in the agroforestry systems practised at each site (Fig. 2).

Liberoid coffee that grows in these locations has been cultivated for a long time using different agroforestry patterns. Area studies are determined by the purposive sampling method, which means that the selection of research locations is done deliberately by considering the research object according to the research objectives to be achieved. This method is used based on considerations of the different characteristics of the locations where Liberoid coffee grows on both islands.

### Data Collection

Environmental observations were conducted by measuring ecological and morphological factors. The ecological factors observed at each location include altitude, latitude, air temperature, air humidity, light intensity, rainfall, soil profile, characteristics and vegetation analysis. Those microclimatic factors at each coffee plantation site are measured and then compared. Altitude and latitude are measured using Essential GPS, temperature and humidity are measured using data loggers (Brand), and light intensity is measured using a digital lux meter (Benetech GM1010). Average monthly temperature, relative humidity and rainfall information is obtained from the local Meteorology, Climatology and Geophysics Agency (BMKG). Phenotypic morphological observations were measured quantitatively and qualitatively based on modified guidelines. The morphological phenotype of Liberoid coffee was observed based on four main groups, namely plant architecture, leaves, fruits and seeds. The details of the observed variables are explained as follows.

### Soil Study

The identification of soil characteristics at all locations is conducted by digging a profile at a depth of 0 cm–200 cm below ground level. Soil profile observations are based on the morphological characteristics of the soil profile, including layer depth (cm), horizon boundary, texture, colour, structure shape, structure size, consistency, plasticity and special characteristics (concretion and rust). Soil samples were taken from the profile, both whole and incomplete soil samples. Soil samples were taken using the diagonal method (Soemarno 2020). The soil samples taken at each horizon were chemically analysed in the laboratory. The parameters and methods used in the soil sample test are presented in Table 1. The morphological soil data and laboratory tests were tabulated and then analysed using the matching method using the Soil Taxonomy Key document from FAO 2014 to determine the classification of the core horizon and its characteristics.

### Morphology Study

The Liberoid phenotypic study of coffee diversity focuses on morphological and agronomic traits. Direct observation used to observe various physical characteristics of plant architecture, leaves, fruits and seeds using descriptors modified from the main reference Descriptors for Coffee (Coffea spp. and Psilanthus spp.) published by the International Plant Genetic Resources Institute (IPGRI) and the Guidelines for the Conduct of Tests for Distinctness, Uniformity and Stability: Coffee, published by the International Union for the Protection of New Varieties of Plants (UPOV).

The variables in the four groups of morphological characteristics analysed include:

1. Architecture Characteristic: Tree shape, plant height (m), trunk circumference (cm), canopy width (m), branching architecture, internode distance of the trunk (cm), internode distance of the branches (cm), local recognition of habitus and age (years).
2. Characteristic: Young leaves colour, mature leaves colour, leaf shape, shape of leaf tip, leaf edge wave, stipule shape, pilosity of domatia, average leaf length (cm), average leaf width (cm), average petiole length (cm), leaf length range (cm), leaf width range (cm), petiole length range (cm).
3. Fruit Characteristic: Fruit shape, fruit colour, fruit stalk, fruit disc shape, average fruit length (mm), average fruit width (mm), average pulp thickness (mm), fruit length range (mm), fruit width range (mm) and pulp thickness range (mm).
4. Characteristic: Seed shape, average seed length (mm), average seed width (mm), average seed thickness (mm), seed length range (mm), seed width range (mm), seed thickness range (mm) and seed colour.

Observations are made using tools such as modified observation forms, labels, sewing tape measures, digital scales, digital calipers, stationery, cameras and Royal Horticultural Society (RHS) Colour Charts.

### Statistical Analysis

In this study, there were quantitative and qualitative data groups based on observations of morphological characteristics, which is plants architecture, leaves, fruits and seeds. The data groups were then converted into numerical values for statistical analysis. The diversity analysis of *Coffea liberica* sensu lato phenotypes was carried out using Principal Component Analysis (PCA) based on Pearson’s correlation matrix, which aims to identify variability patterns and reduce data dimensions without losing significant information. The main components that contribute to the total variability are determined based on eigenvalue > 1, which is analysed using XLSTAT software version 2024.3. The characters that have a significant contribution to morphological variability are determined based on loading factors > 0.6. This shows that corresponding morphological character is the main determinant in the morphological variation of Liberoid coffee.

The next data analysis is to identify the hierarchical relationship between Liberoids coffee phenotypes using Agglomerative Hierarchical Clustering Analysis (AHC). Grouping method that is used in AHC analysis is the Unweighted Pair Group Method with Arithmetic Mean (UPGMA) with a similarity distance measure based on the Pearson correlation coefficient. The dendrogram resulting from AHC illustrates the pattern of genotype grouping based on phenotypic similarity. Cluster cutting is done at a similarity level determined based on significant changes in the clustering structure. This cluster separation is used to compare the Liberoid coffee population from three research locations (Jember, Jombang and Jambi) and to determine phenotype groups that have similar characteristics.

## RESULTS

### Soil Conditions and Environmental Factors

#### Soil factors

The location of this study is in three Liberoid coffee agroforestry areas with contrasting ecological landscapes, namely Betara (Jambi), Wonosalam (Jombang) and Silo (Jember) Districts. The main objective is to show how the soil and environmental conditions at each location affect the phenotypic appearance (physical characteristics) of Liberoid coffee.

Betara (Sumatera) is located in lowland peatlands with organic soil typical of ustifolists which is characterised by a dusty clay texture (sand 2%, dust 66%, clay 32%), soil pH is very acidic (3.5–4.5), and water saturation due to poor natural drainage. These conditions make the plant adapt to high acidity and limited nutrient availability because most nutrients are trapped in difficult organic matter decompose.

Meanwhile, Wonosalam and Silo (Java) both have Inceptisols soil types with the typical dystrudepts class, which is soil that develops in the moderate weathering phase. The soils in both areas have striking differences in texture and drainage. Wonosalam (Jombang) has a clay clay textured soil with dusty clay (sand 5%, dust 63%, clay 32%) with a pH ranging from 5.25–6.51, while Silo (Jember) has a clay clay texture with a dusty clay texture (sand 8%, dust 50%, clay 42%) with a pH that tends to be more neutral at 6.59–6.91.

In general, the main differences between soils in Sumatra (Betara) and Java (Wonosalam and Silo) lie in soil type (organic vs. mineral), acidity level (very acidic vs. slightly acidic–neutral) and drainage conditions (water saturated vs. good). This has a significant impact on soil fertility conditions (Fig. 3).

#### Ecological factors (microclimate conditions)

Ecological factors are the fundamental foundation that affect the growth and development of plants in an area. This study observed the environment of growing Liberoid coffee in three locations that have different agroforestry and microclimate ecosystems. The results of the observations show that there are significant variations in terms of elevation, air temperature, humidity, sunlight intensity and topographic characteristics, as well as other types of plants that make up agroforestry (Fig. 4).

The results of observations in Mekar Jaya Village, Betara District (Jambi) have a low elevation ranging from 15 to 21 metres above sea level (masl) with a high air temperature of around (range) 32°C, very high humidity (85%) and low sunlight intensity (4,349 lux). This area is a peat lowland that tends to be flat with a type C (Commercial Polyculture) agroforestry system. Liberica coffee plants are planted together with areca nut (dominant), oil palm and coconut in limited quantities.

Meanwhile, Mangirejo Hamlet, Wonosalam Village (Jombang), is located in a hilly area in the Anjasmoro Mountains with a higher elevation, around 600–700 masl. This location has cooler temperatures (26°C), moderate air humidity (80%) and medium sunlight intensity (14,093 lux). This area is classified as a more complex type B (Traditional Polyculture) agroforestry, with a combination of shade trees in the form of durian, avocado, mango, bananas and ginger.

Different conditions are clearly seen in Mulyorejo Village, Silo District (Jember), which is located at a medium elevation, around 350–425 masl. The environment in this region has a high air temperature (32°C), the lowest air humidity (65%) and a very high intensity of sunlight (45,357 lux). This area is a hot hill with type C (Commercial Polyculture) agroforestry that lacks shade after sengon trees are harvested. Other plants that grow side by side with Liberica coffee in this location include mahogany, with a dominance of robusta coffee plants.

The differences in soil conditions and environmental factors from the three diverse Liberoid coffee growing locations form a unique ecosystem. Liberoid coffee has a wide degree of adaptability, which encourages a level of phenotypic diversity based on genetic and environmental interactions. Soil conditions and environmental factors from the three sites are assumed to play a role in the phenotypic variation of Liberoid coffee grown in agroforestry systems. The results of observations show that Liberoid coffee is able to survive in extreme conditions. This potential can make Liberoid coffee an important candidate in overcoming marginal land management.

### Morphological Factor

Morphological factors are the physical characteristics of plants that can be observed. The morphological factors of Liberoid coffee were observed qualitatively and quantitatively. The morphological characters observed include plant shape, leaf character, fruit character and seed character. These morphological variations will then be processed using PCA to find key morphological markers in the variability of the Liberoid phenotype grown in three different locations.

Principal Component Analysis is used in this study to simplify and explain the phenotypic variation patterns of Liberoid coffee plants clearly. Starting from the Pearson correlation matrix which describes the relationships between plant phenotypic characters, it is then calculated as the basis for determining the most relevant key components. In simple terms, eigenvalue is a number that indicates how much information (phenotype diversity) is described by each component in the PCA. The greater the eigenvalue, the greater the importance of the component in describing phenotypic data variations (Fig. 5).

### PCA result

### Principal Component Analysis (PCA)

In the phenotypic data, Liberika coffee produced a total of 29 components (F1 to F29), each of which represented the dimension of diversity in the data. However, not all components have statistically significant contributions. Based on eigenvalues, only the first eight components (F1–F8) have eigenvalues greater than 1 and are generally considered relevant for further analysis. The first component (F1) explains 30.49% of the total diversity, followed by the second component (F2) at 11.61%, and F3 at 10.04%. Meanwhile, the total cumulative diversity up to the eighth component (F8), which was explained, has reached 79.62%. Outside of those eight components, the eigenvalue is below 1, which means its contribution to total diversity is very small and is conventionally not used in the main interpretation.

The results of the factor loadings analysis in the PCA provide a deeper understanding of what phenotypic variables contribute the most to each of the main components (F1–F5). Factor loadings reflect the strength and direction of the relationship between each variable and the main component. A high value (positive or negative) indicates that the variable strongly determines the formation of the dimension.

The second component (F2) is dominated by the vegetative character of the plant, such as plant height (0.70), trunk girth (0.70), diameter of canopy (0.72), and young leaf color (0.66). This indicates that F2 is a dimension that describes the overall diversity of plant size and structural performance, which may be closely related to age, growth strength, or plant response to different agroecological conditions. The third component (F3) although describing a smaller proportion of diversity, specifically accommodates characters related to fruit shape (0.75), fruit disc shape (0.32), and seed shape (0.29). This component can be understood as a representation of the morphological shape of the fruit and seed, not its size. The fourth component (F4) shows a strong contribution of leaf apex shape (0.64) indicating that the micro-morphological aspects of the leaf also have informative weight in phenotype diversity. While the fifth component (F5) has no character with a loading factor value of > 0.6.

Interpretation of the results shows that the main diversity of Liberoid coffee plants in the agroforestry landscapes of Sumatra and Java is most explained by the character of the fruit and seeds (F1). Furthermore, the size and vegetative structure of plants (F2) and aspects of form and morphological detail (F3–F5) also contribute to this diversity. These findings confirm that not all phenotypic characters have the same role in shaping the phenotype variation of Liberoid coffee.

#### Cluster modelling (dendogram)

Agglomerative Hierarchical Clustering analysis was used in this study to understand the phenotypic similarity patterns of *C. Liberica* sensu lato plants from various agroforestry locations in Sumatra and Java. This approach results in groupings based on the level of phenotypic similarity visualised through a dendrogram. The AHC analysis succeeded in grouping 31 *C. liberica* sensu lato accessions from three different agroforestry locations: SLO (Silo, Jember), WSL and MGRJ (Wonosalam, Jombang), and TJB (Betara, Jambi). The results of the grouping formed five main clusters that reflect phenotypic similarities between individuals based on morphological characteristics (Fig. 6).

The first cluster (1) in blue was the largest group with 18 individuals, which included most of the genotypes from SLO, WSL, and MGRJ. This group also includes several individuals from TJB, such as TJB-PL04 and TJB-BYT 1. This composition shows that although genotypes come from different geographical locations, there is a fairly high phenotype similarity, especially between coffee from the agroforestry ecosystem of Java (Jember and Jombang). However, this class also has a high level of internal cluster diversity (within-class variance = 529,432), indicating that there is still considerable phenotypic variation within this group.

The third (3) cluster in green contains eight individuals, almost all of whom are from Jambi (TJB) and one from Wonosalam (WSL-03). The phenotypic characteristics of these genotypes are relatively uniform and different from other genotypes. Meanwhile, the fourth cluster (4), which consists of only three individuals (TJB-PT1, TJB-PT2, TJB-PT3), also comes from Jambi and forms a separate group that is relatively homogeneous. This means that Liberika coffee from Jambi tends to form a different and more stable phenotypic subpopulation, showing a tendency to homogeneity within the region.

Interestingly, class 2 (SLO-3) and class 5 (TJB-PT5) consist of only one individual. This suggests that the two genotypes are very different from each other and can be considered outliers in the population. SLO-3 from Jember and TJB-PT5 from Jambi exhibit unique phenotypic expression and are most likely influenced by a specific combination of local genetics and extreme agroecological conditions. This phenomenon could be an indication of the presence of environmental pressures or specific adaptive responses that form the unique phenotype. These two cultivars are important to review further because they may hold superior potential traits that are not found in other groups.

These results show that the phenotypic diversity of Liberika coffee in Sumatra (Jambi) is not homogeneously distributed but is spread in several different small groups. These conditions of Jambi plants show more varied phenotypic expressions, possibly influenced by composite sources of planting material. On the other hand, genotypes from the Java region (Jombang and Jember) show the opposite pattern. Most of the individuals from the two different locations in Java are members of one large cluster that is consistent, illustrating a strong phenotypic homogeneity despite coming from different districts.

## DISCUSSION

### Phenotypic in Historical Approach

The exploration of phenotypic diversity in *Coffea liberica* accessions in Indonesia is inseparable from its intricate taxonomic history. Ambiguity in naming and morphological identification of this coffee has long been debated among scholars (Bridson 1982; Cramer 1957; Hiern 1876). Initially, the name “liberica” was not intended as a scientific designation but served as a geographic identifier for coffee seedlings obtained from the west coast of Africa in the early 19th century (Hiern 1876). Furthermore, Bridson indicated that early research on this coffee type was still in its preliminary stages (Bridson 1982; 1994). Collections and seedlings gathered by several researchers at the time had not undergone selection or breeding processes (Hiern 1876). Nevertheless, this coffee was widely disseminated by 1873 to combat leaf rust devastating *C. arabica* (Burgers 2010; Cramer 1957).

The hasty introduction of *C. liberica* seedlings led to ambiguity regarding the genetic origins of the cultivated material. As a result, historical records of early introductions in Java, Ceylon and other colonies consistently documented broad morphological variability (Cramer 1957; Nicholls 1881; Thurber 1881). This extensive variability prompted many researchers to report various forms (Bridson 1982; Cramer 1913). Therefore, Cramer employed the term “Liberoid” to categorise coffee variants resembling or related to *Coffea liberica i*n taxonomic and breeding selection research in Indonesia (Cramer 1957).

Research on Liberoid coffee in Indonesia ceased for an extended period due to political circumstances at the time. This study seeks to reconnect Cramer’s knowledge legacy with the observed diversity of field accessions. Phenotypic diversity documented across Sumatra and Java landscapes is interpreted as the dynamic outcome of a century of adaptation and represents direct descendants of populations recorded by Cramer during that era. This process aligns with the ecological principle that morphological richness emerges from interactions between genetically diverse populations and numerous micro-environmental niches (Clifford & Willson 1985).

A key limitation of this study is that although recent molecular studies (Davis *et al*. 2025) have confirmed *C. liberica, C. dewevrei* and *C. klainii* as distinct species, the local Indonesian accessions examined have not yet been integrated into the reference genetic database. Consequently, the term “Liberoid” is retained to describe the observed coffee populations, while acknowledging that the specific identity and phylogenetic relationships of these populations require further confirmation through comparative analysis with established genetic reference data.

### The Role of Soil Conditions in Determining Phenotype Variation of Liberoid Coffee

This study underscores the importance of soil conditions as the main foundation that controls the phenotypic variation of Liberoid coffee plants. The results of observations in Betara (Jambi) show that peatlands with a very acidic pH (3.5–4.5), high organic matter content and difficult mineralisation create barriers to access to nutrients for plants (Harenda *et al*. 2018; Maryani & Novriadhy 2022). These conditions make plants have to adapt to high acidity and limited nutrient availability because most of the nutrients are trapped in hard-to-decompose organic matter (Bourdon *et al*. 2021; Maryani & Novriadhy 2022; Santi *et al*. 2022).

This fact forces Liberoid coffee to adapt through the character of the smaller but thick-pulped fruit, which is one of the physiological strategies to survive in extreme environments (Bakri *et al*. 2018). In contrast, the soil of Inceptisols (Typic Dystrudepts) in Wonosalam (Jombang) and Silo (Jember) has a more neutral pH (5.25–6.91), as well as a clay-dusty structure that facilitates aeration and nutrient absorption (Carréra *et al*. 2023). Liberoid coffee plants in this location tend to show faster vegetative growth with wider leaves, sturdier stems and an expanded canopy. These results are in line with studies that confirm that a slightly acidic–neutral pH is ideal for coffee (Eddin et al. 2024). The morphological variation of Liberoid seeds observed across the three agroforestry landscapes as shown in Fig. 7.

### The Influence of Microclimate and Agroforestry Patterns on Phenotype Adaptation

The combination of air temperature, humidity and light intensity at all three locations encourages striking phenotypic diversity. The condition of Betara (Jambi) with relatively high humidity (> 80%) and low light intensity (± 4,349 lux) makes plants more efficiently absorb and utilise light. This environment gives rise to relatively thin leaf characters and more stomata, adjusting to the minimal light supply (Assis *et al*. 2019; Carréra *et al*. 2023). Wonosalam (Jombang) is located at a medium elevation (600–700 masl) with moderate temperatures (26°C) and complex agroforestry patterns, so that Liberoid coffee can photosynthesise optimally without excessive light stress. In Silo (Jember), the plant faces hot temperatures (32°C) with the lowest humidity (65%) and the highest light exposure (± 45,357 lux). The lack of shade trees causes coffee leaves to thicken the palisade tissue, to suppress evaporation and prevent water exhaustion (Adi *et al*. 2019; Davis *et al*. 2021; Fuchs *et al*. 2021). This diverse adaptive response is in line with the theory that agroforestry composition, elevation and light are key factors that shape the morphological character of coffee plants (Breitler *et al*. 2022; Farah 2019; Gomes et al. 2020). Such variation is evident in the bush, tree and shrub growth forms of Liberoid coffee observed in the field (Fig. 8).

### Significance of PCA Results on Phenotype Diversity

PCA shows the dominance of fruit-seed characters (F1) in explaining the phenotypic diversity of Liberoid coffee, followed by vegetative size (F2 and F3). The prominent character of the fruit and seeds reflects a high sensitivity to environmental influences, especially regarding size, weight and pulp. The character of fruits and seeds is the main determinant in the assessment of potential genotypes for coffee agroforestry. This aspect is a priority for breeding because it is directly related to productivity and crop quality (Gomes *et al*. 2020; Hafif *et al*. 2024). On the other hand, the magnitude of the influence of vegetative character confirms that nutrient availability and stability of environmental conditions greatly impact plant size. Adaptive variety selection efforts can make both character groups (fruit-seed and vegetative) the main indicators, depending on the cultivation goal (marginal vs. intensive areas) (Chevalier 1946; Lambot *et al*. 2017).

### Clustering Between Regions: Sumatera vs. Java

The diversity of phenotypes in Jambi is divided into several small groups, indicating a high level of heterogeneity. Liberoid coffee plants in peatlands appear to come from different genetic sources or are formed due to microhabitat variation (Dougherty 2012; Hakim 2021; Nair *et al*. 2021; Pasandaran *et al*. 2015). Meanwhile, in the Java region (Jombang and Jember), most of the Liberica coffee individuals join a large group that is relatively homogeneous. This phenomenon indicates that coffee plantations in Java are adopting a more uniform agroforestry system, such as the use of similar shade trees and standard agricultural practices, thus giving rise to similar plant morphological characteristics across locations (Adi *et al*. 2019; Fuchs *et al*. 2021; Lew 2022). The presence of one or two outlier genotypes (e.g. SLO-3 or TJB-PT5) indicates unique genetic potential that is suitable as a source of gene diversification (Arimarsetiowati *et al*. 2023; Belletti *et al*. 2017; Depecker *et al*. 2023; Hendre et al. 2008). These outliers are clearly separated from the main cluster of accessions in the principal component biplot (Fig. 9).

### Future Research Challenges and Directions

This study has revealed the adaptation pattern of Liberoid coffee in the agroforestry landscape of Sumatra and Java. However, a more comprehensive understanding requires an exploration of the genetic aspects underlying phenotypic variability and multilocation assays on other marginal lands (Colfer & Prabhu 2023; Hakim *et al*. 2022). The relationship between phenotype, secondary metabolite content and coffee flavor has not been fully explored, so further molecular and chemical approaches are needed. Further research is also recommended to test the plant’s response under climate change pressures, such as extreme temperature rise and pathogen attacks, so that Liberoid coffee can be an important component in future adaptation strategies (Alotaibi 2023; Legesse 2019; Shrestha *et al*. 2022).

## CONCLUSION

This study confirms that Liberoid coffee has a high phenotype diversity that is strongly influenced by differences in agroecological conditions, especially soil type and microclimatic factors. Coffee grown in the peatlands of Jambi (Betara) adapts to producing smaller fruits but thicker pulp, while plants in Javanese mineral soils (Wonosalam and Silo) show a larger vegetative morphology with more developed crown and leaf structures. The results of the PCA showed that the character of fruit and seeds was the main determinant in explaining phenotype variation, followed by vegetative character. Cluster analysis (AHC) reinforces the finding that the Jambi population has high heterogeneity within its region, while the Javanese population is more homogeneous but still retains a unique genotype. Overall, these results confirm the need to conserve the genetic resources of Liberoid coffee in order to strengthen plant resilience and breeding adaptive varieties in agroforestry lands, especially in the face of climate change challenges and the need for diversification of plantation commodities.

### Research Implications for Conservation on Agroforestry and Liberoid Coffee Breeding

The discovery of high phenotypic variation in Sumatran peatlands has implications for the rich genetic conservation potential. Genotypes with unique adaptations to acidic pH or low nutrient availability are likely to be used to improve plant resilience in marginal land (Gillson *et al*. 2020; Rozzi *et al*. 2023). The existence of homogeneous clusters in Java can be the basis for the development of stable varieties with consistent morphological characteristics for a more “controlled” agroforestry system. Breeding programs can utilise the results of PCA to select priority superior characters, such as fruit size, pulp thickness, and plant height, according to market needs and land conditions (Arimarsetiowati *et al*. 2023; Baltazar & Buot 2019; Fardinatri *et al*. 2024; Joshi 2023). The diversity found reinforces the urgency to preserve local coffee genetic resources as a basis for breeding and crop resilience to climate change on agroforestry land. These results also serve as the basis for the integration of Liberoid coffee species in agroforestry diversification and local germplasm conservation (Charnley *et al*. 2007; Faronny *et al*. 2023; 2024; Hariyati *et al*. 2022).

## Figures and Tables

**FIGURE 1:**
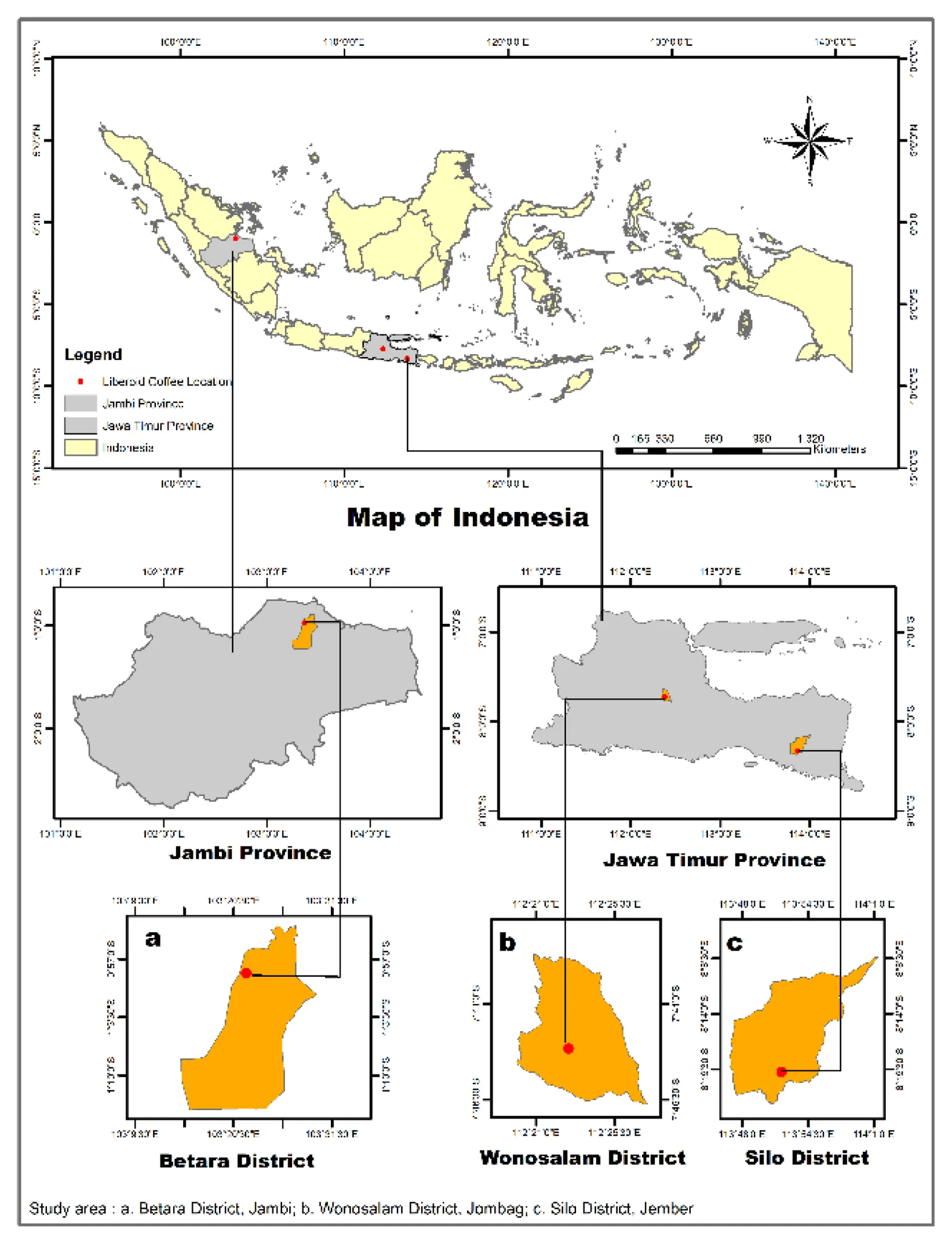
Study area: Batara (Sumatera), Wonosalam and Silo (Java).

**FIGURE 2:**
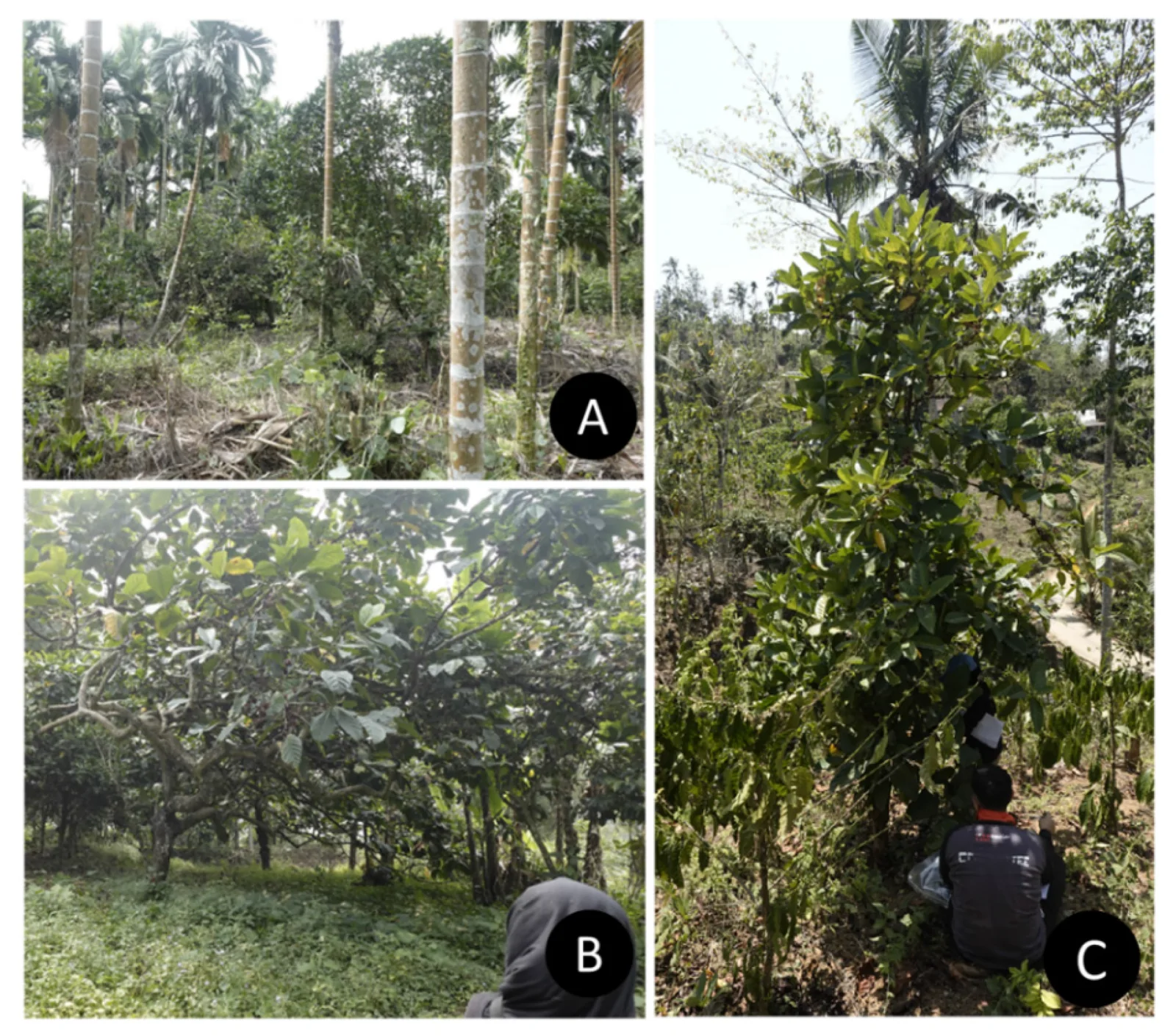
Agroforestry in three locations. (A) Batara; (B) Wonosalam; (C) Silo.

**FIGURE 3:**
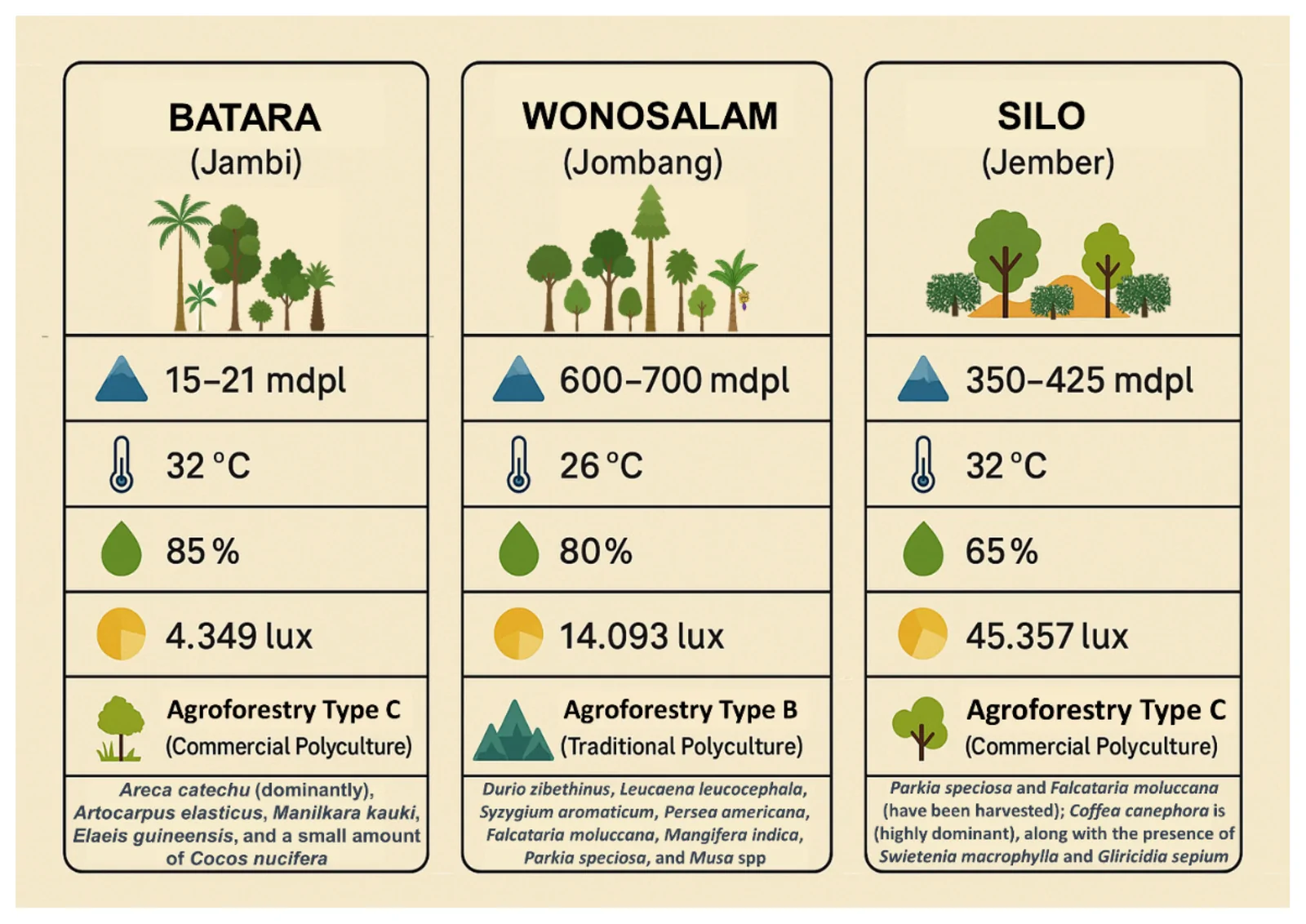
Agroecological characteristics of coffee-based agroforestry landscapes.

**FIGURE 4:**
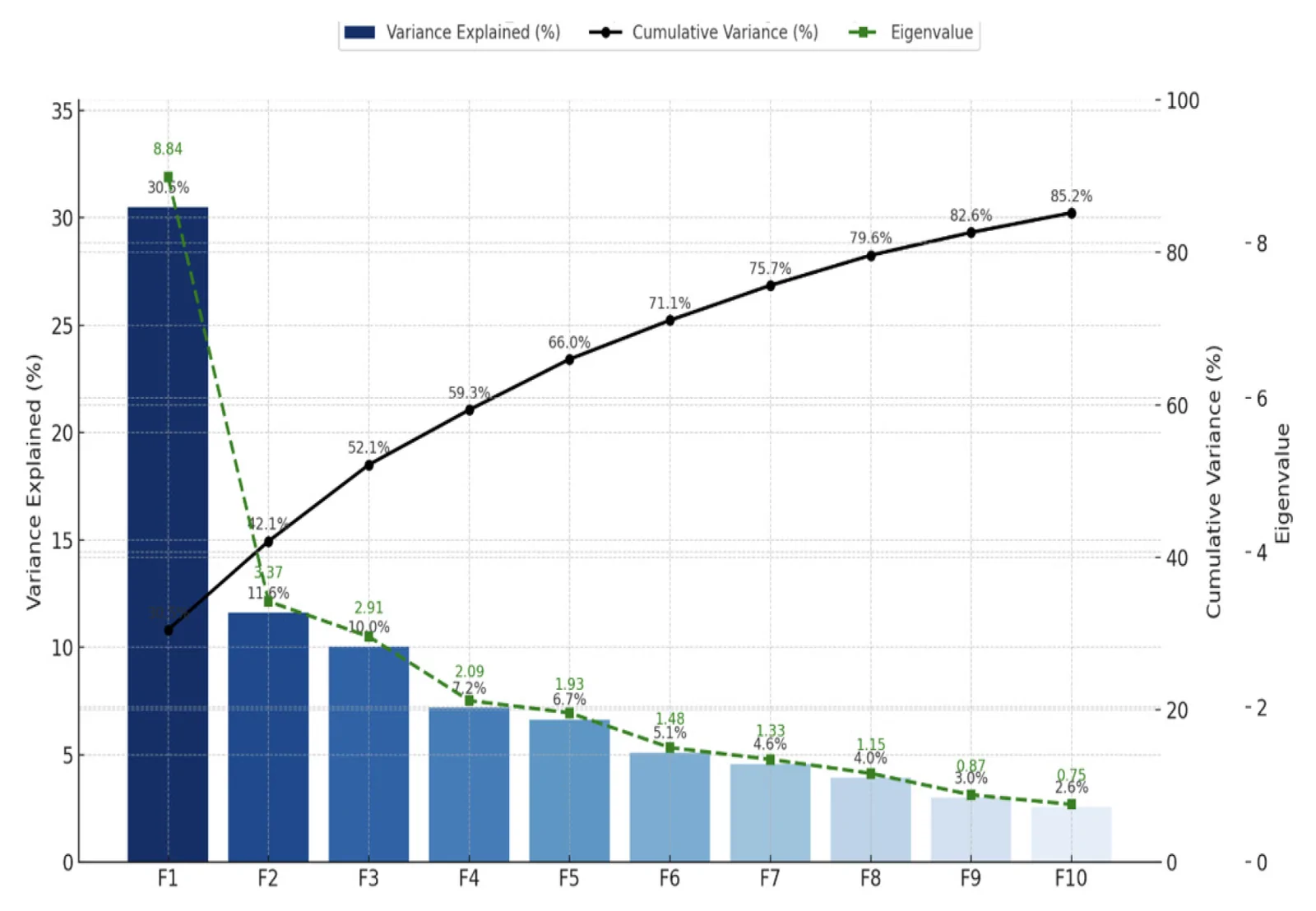
Results of the factor analysis.

**FIGURE 5:**
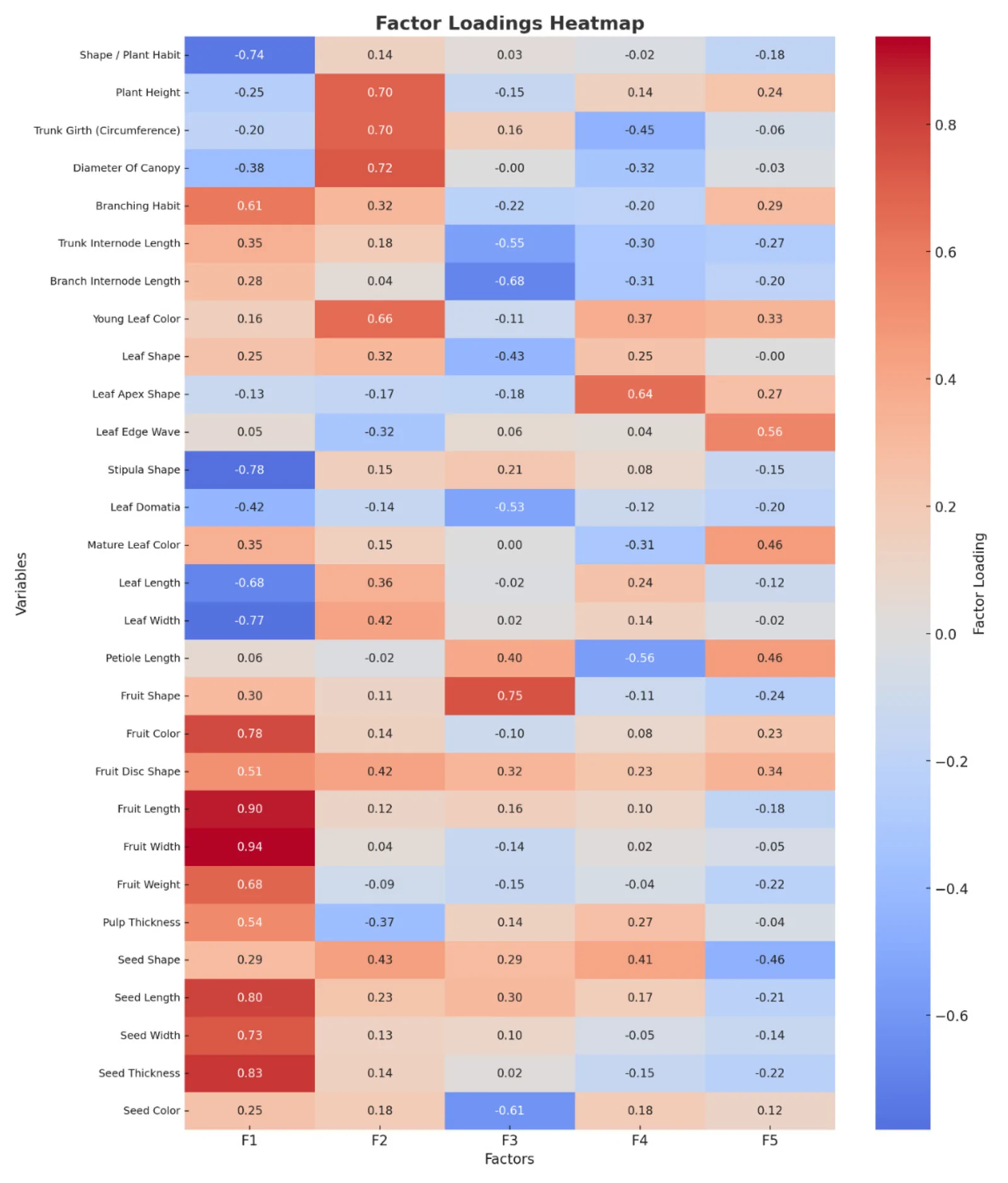
Factor loadings heatmap of morphological traits.

**FIGURE 6:**
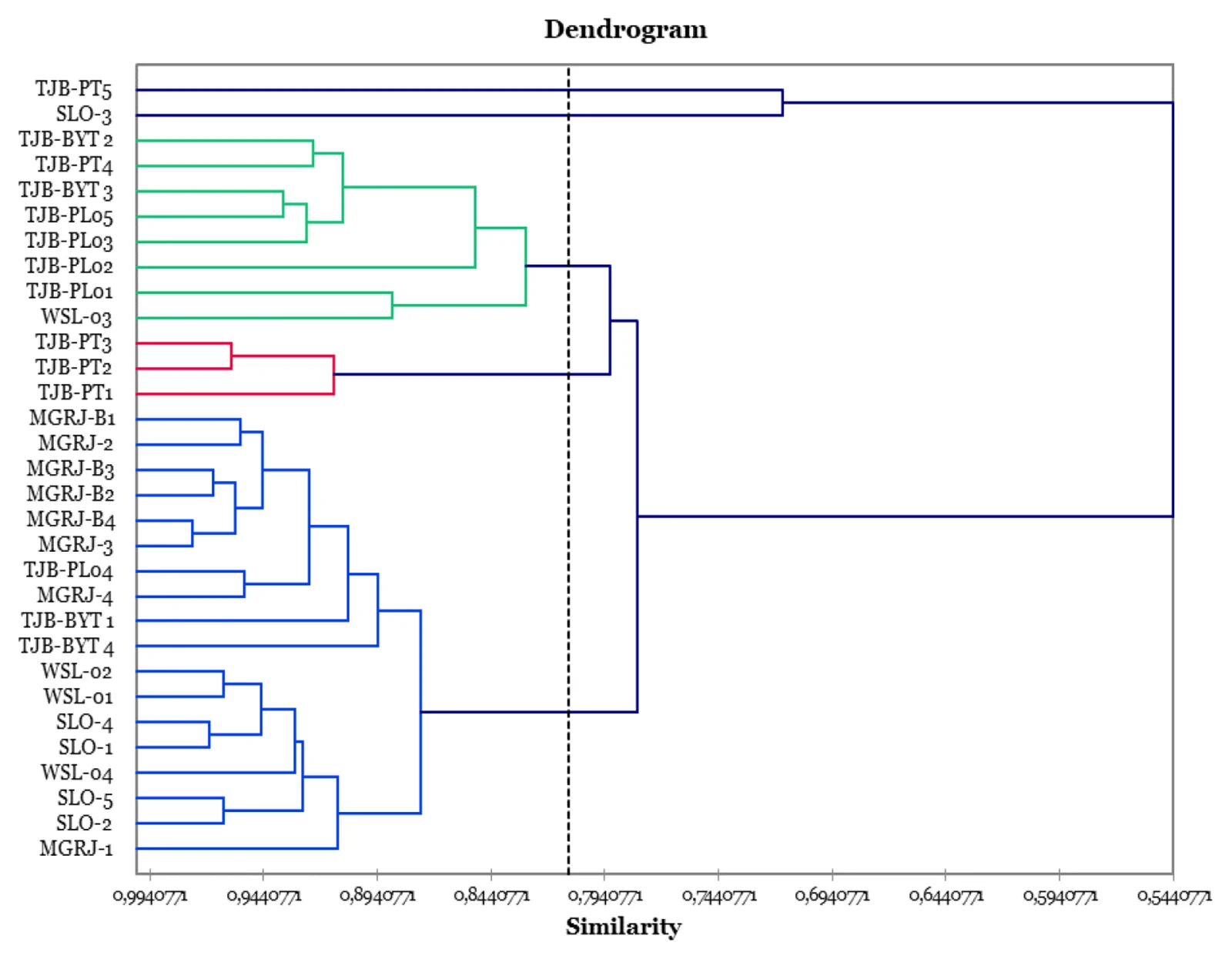
Dendrogram of phenotypic similarity among C. liberica sensu lato accessions based on AHC.

**FIGURE 7:**
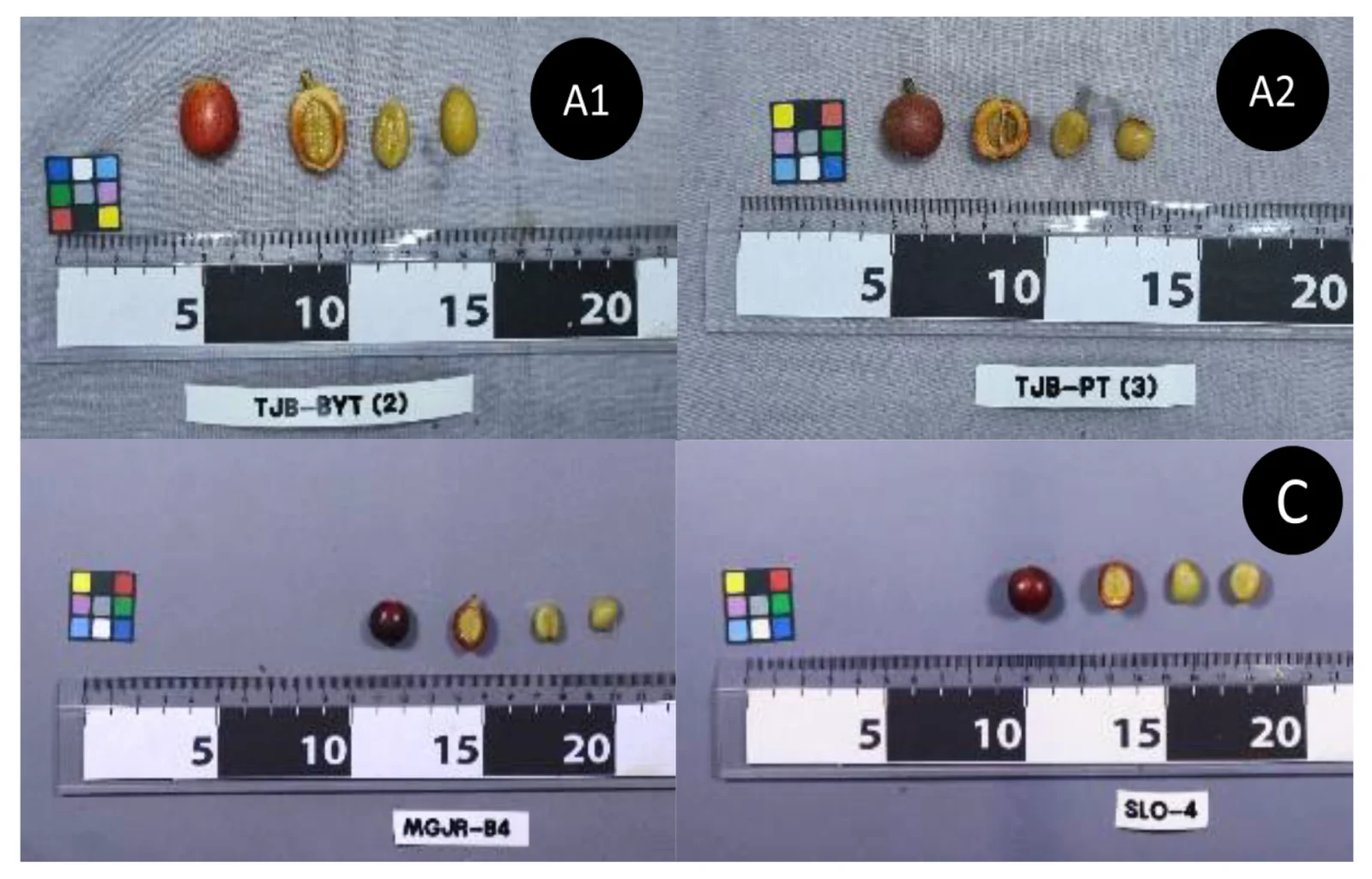
Representative morphological variation of Liberoid seeds from different agroforestry landscapes: (A1–A2) Batara (Jambi) presume *C. liberica*; (B) Wonosalam (Jombang) presume *C. dewevrei*; and (C) Silo (Jember) presume *C. dewevrei*.

**FIGURE 8:**
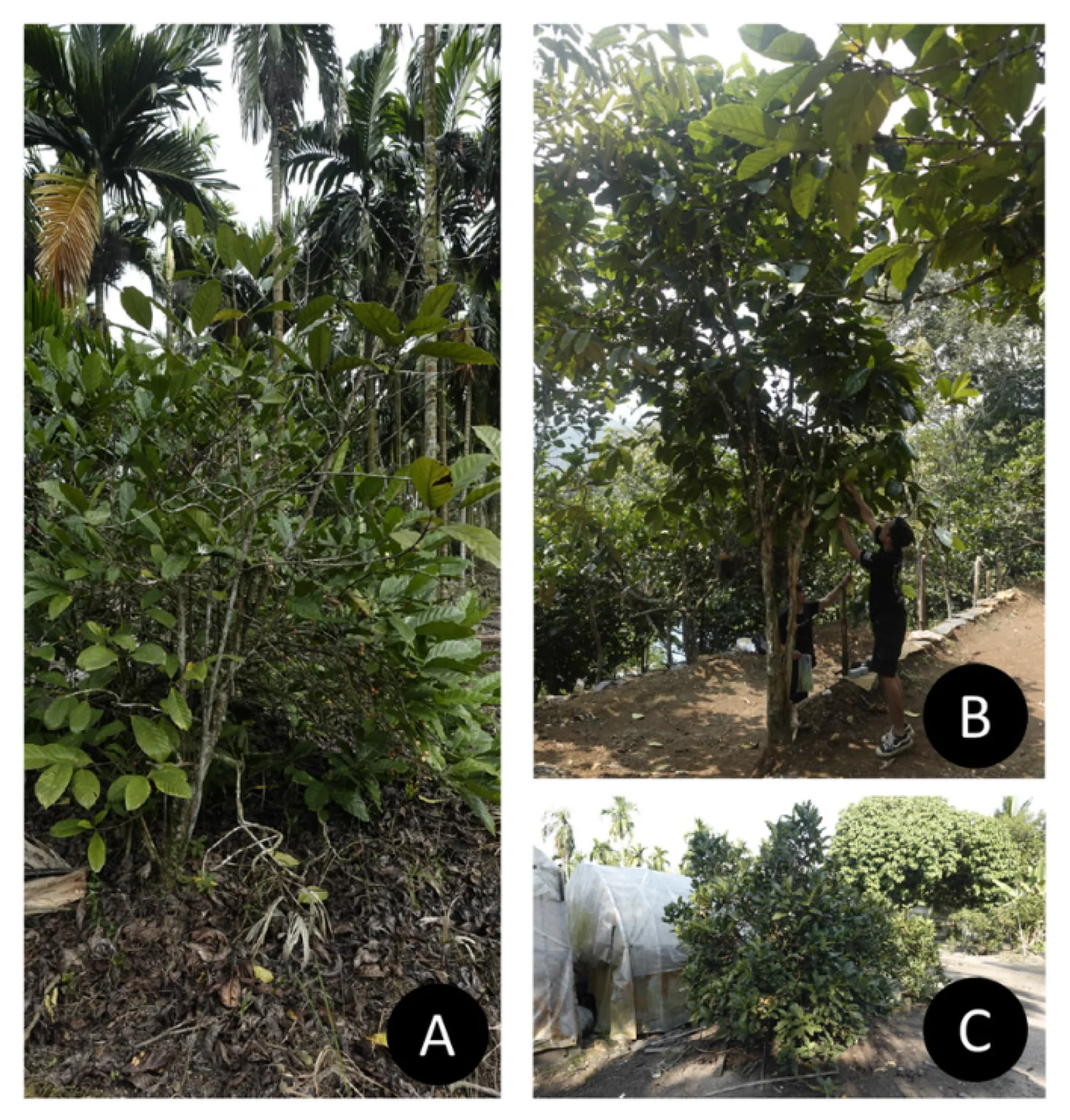
Growth forms and field habitat of Liberoid coffee (Pachycoffea). (A) bush; (B) tree; and (C) shrub.

**FIGURE 9:**
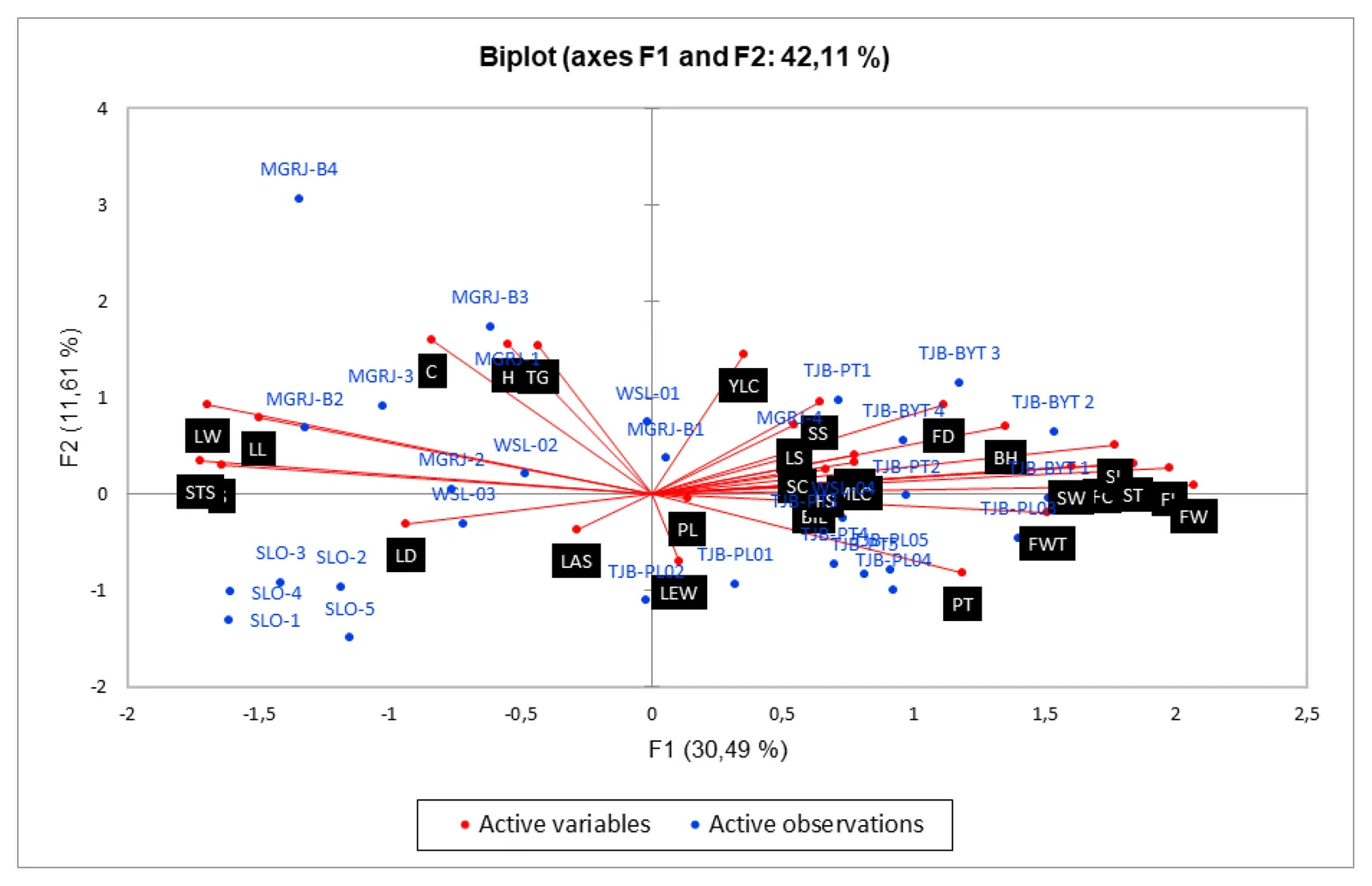
Principal component biplot of Liberoid coffee phenotypes based on F1 and F2 (42.11%) axes showing the distribution of observed accessions and contribution of morphological variables.

**TABLE 1:** Parameters and methods of soil sample testing in the laboratory.

Parameter	Methods	Outputs
C-organic	Walkey and Black	Organic component
Texture Mineral Soil	Pipette	Percentage of soil particles
Texture and Peat	Soil Depotition	Peat Soil Maturity Level
Soil Colour	Methode and Musell	
	Soil Colour Chart	
Soil pH	pH Meter	Soil Acidity Level
Friability	Bulk Dencity and Capacity	Level of Looseness, density and porosity of the soil
N total	Kjeldahl	Nitrogen Level in Soil
P2O5	Olsen and Bray	Phosphor Level in Soil
K2O	Morgan Wolf	Potassium Level in Soil
CEC	Leaching Ammonium acetate	Cation levels can be exchanged in the soil
